# Associations and interactions between *APOE* e4 genotype and lifestyle with brain structural phenotypes

**DOI:** 10.1093/braincomms/fcaf350

**Published:** 2025-09-16

**Authors:** Yating You, Joey Ward, Frederick K Ho, Donald M Lyall, Claire E Hastie

**Affiliations:** School of Health and Wellbeing, University of Glasgow, Glasgow, Scotland, G12 8TB, United Kingdom; School of Health and Wellbeing, University of Glasgow, Glasgow, Scotland, G12 8TB, United Kingdom; School of Health and Wellbeing, University of Glasgow, Glasgow, Scotland, G12 8TB, United Kingdom; School of Health and Wellbeing, University of Glasgow, Glasgow, Scotland, G12 8TB, United Kingdom; School of Health and Wellbeing, University of Glasgow, Glasgow, Scotland, G12 8TB, United Kingdom

**Keywords:** lifestyle, brain structure, MRI, NODDI, *APOE* e4

## Abstract

Apolipoprotein *E* gene (*APOE*) e4 allele is known as the strongest common genetic risk factor for Alzheimer’s disease. Various lifestyle factors are reported to have multiple associations with brain health, including smoking, alcohol intake, diet, sedentary behaviour and physical activity. However, the associations and interactions of overall or individual modifiable lifestyle factors and *APOE* e4 genotype, on brain volumes and white matter tract integrity measured by diffusion tensor imaging and neurite orientation dispersion and density imaging (NODDI), remain incompletely understood. In this cross-sectional study, we used data from the UK Biobank MRI assessment (*N* = 24 912) participants living without dementia. They were classified as having ‘favourable’, ‘moderate’ and ‘unfavourable’ levels based on their lifestyle scores (0–10 points) being calculated by the magnitudes of adherence to five healthy lifestyle guidelines: two points for each healthy category, one point for moderate ones, and zero point for unhealthy ones. Neuroimaging markers, namely brain volumes and white matter tract integrity measures, were derived from MRI. Linear regression was performed to examine the associations and interactions of lifestyle levels, *APOE* e4 genotype and each lifestyle factor, with multiple brain structural MRI phenotypes. We found that compared with favourable-level lifestyle, moderate and unfavourable levels were significantly independently associated with smaller grey matter, hippocampus and total brain volumes (standardized β range 0.004–0.096 per unit). Significant associations were found between *APOE* e4 presence and smaller hippocampal volumes (β range 0.042–0.048 lower versus absence) and worse white matter tract integrity. Individual unhealthy lifestyle aspects were mostly associated with poorer brain structural markers, such as current smoking (β range −0.014 to 0.209), high-level drinking (β range −0.105 to 0.152) and grey matter and white matter, as well as both traditional white matter tract and NODDI metrics, in both individually and simultaneously modelled lifestyle-factor models. The interactions between lifestyle levels and *APOE* e4 status on brain metrics were generally non-significant. In conclusion, the presence of *APOE* e4 and non-favourable lifestyles were mostly associated with worse brain health, and this was largely linear rather than synergistic. The benefit of a healthy lifestyle on brain health does not vary by genetic risk for dementia.

## Teaser

You *et al*. reported among older adults that *APOE* e4 genotype and non-favourable lifestyles were associated with smaller brain volumes and poorer white matter tract integrity. No interactions were found between lifestyle and *APOE* e4. Healthy lifestyle benefits brain health regardless of genetic risk for dementia.

## Graphical Abstract

**Graphical Abstract:**
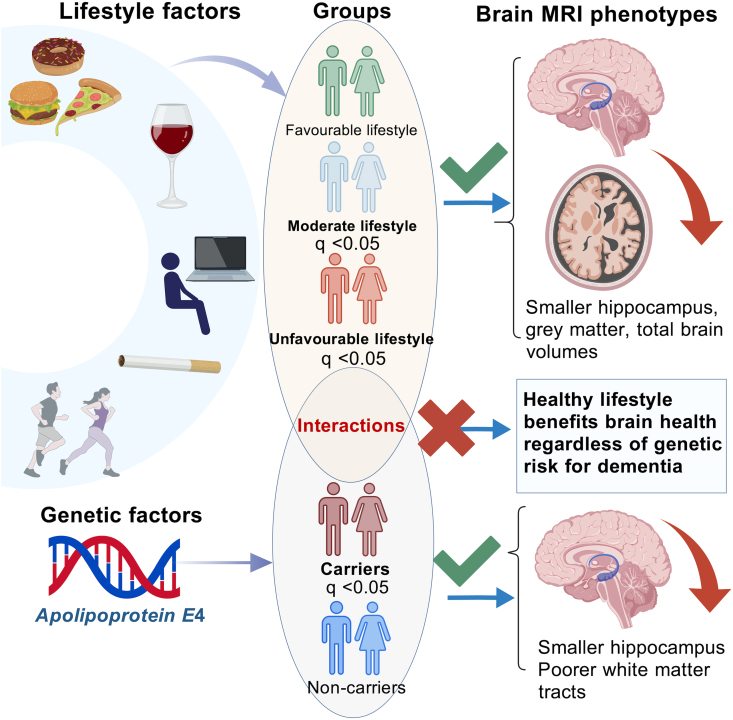

## Introduction

Dementia leads to progressive deterioration in cognition function, along with ability loss in daily activities, and is the seventh leading cause of death globally.^1^ Around 50 million people lived with dementia in 2020, and it is estimated to increase to 153 million by 2050.^2^ It increased individuals and their families’ economic burdens where over 1.3 trillion US dollars were spent on treatments and care globally annually.^1^ Yet, no effective treatments are currently available for dementia. Understanding the contributions of modifiable risk factors and formulating appropriate measures is the priorities to prevent dementia.

At the preclinical stage of dementia, neuroimaging brain structural markers conducting as sensitive precursors, are sometimes used to explore risk factors for the aging brain and its mechanisms.^3^ Prior studies demonstrated, for example, smoking is associated with smaller grey matter (GM)^4^ and chronic alcohol use with disruption of white matter (WM) tract integrity,^5^ as well as unhealthy diet with decreased brain volume and connectivity.^6^ These studies reported significant relations between individual lifestyle and brain health measures and gave the insights of the complex effects of overall lifestyle factors on brain health. For example, a cross-sectional study including 19 822 participants in UK biobank showed significant associations between a healthier lifestyle and smaller brain volumes, including total brain, GM, WM, hippocampus and WM hyperintensities—although without considering genetic risk.^3^ Another study reported the same conclusions by utilizing the well-defined healthy lifestyle based on American Heart Association criteria rather than national guidelines among UK participants.^3,7^ Neither study investigated associations with WM tract integrity, and each lifestyle factor was dichotomized, which might lead the associations between lifestyle factors and brain health to be underestimated.

Apolipoprotein *E* gene (*APOE*) e4 allele is known as the strongest common genetic risk factor for Alzheimer’s disease.^8^ Brain structural differences can be observed between carriers and non-carriers of *APOE* e4, and these may be considered markers of pre-symptomatic Alzheimer’s disease years before disease onset.^9^ A potential biological rationale is that the *APOE* locus pleiotropically affects synaptic plasticity, neuroinflammation, lipid transport and metabolism and e4 alle increased risk for cerebral amyloid angiopathy, which is considered to lead to synaptic dysfunction and neurodegeneration in Alzheimer’s disease.^10^ Moreover, *APOE* e4 carriers might be vulnerable to the impact of unhealthy lifestyle factors. For example, among e4 carriers, low-moderate intake of polyunsaturated, and moderate-high intake of saturated fats and physical inactivity were associated with a more pronounced increased risk of dementia/Alzheimer’s disease.^11^ On the other hand, some prior research states that regardless of genetic risk, higher adherence to the Mediterranean-DASH Intervention for Neurodegenerative Delay (MIND) diet was associated with lower dementia risk (i.e. risk does not vary by *APOE* genetic status).^12^ The interactions between *APOE* e4 and lifestyle factors on brain health are therefore not always consistent. It is important to understand to what extent lifestyle factors contribute to brain structural differences in older people without dementia, between carriers and non-carriers of *APOE* e4, which might aid the development of corresponding interventions for better brain health.

Significant associations between risk factors of dementia and WM integrity tract measured by diffusion tensor imaging have been reported, for instance, higher aggregate vascular risk factors and decreased fractional anisotropy (FA) or increased mean diffusivity (MD), indicating poorer brain health; *APOE* e4 presence and increased WM hyperintensity volumes.^13,14^ However, advances in WM integrity measurement—neurite orientation dispersion and density imaging (NODDI) being more sensitive and specific to clinical outcomes and target pathologies than traditional ones—has not been used to investigate the potential joint influence of lifestyle factors and *APOE* e4 genotype.

Generally, the study of lifestyle factors and *APOE* e4 genotype on brain volumetric markers has been explored with potentially underestimated and inconsistent findings. The associations of overall informative lifestyle factors and *APOE* e4 genotype on brain volumes and WM tract integrity—diffusion tensor imaging and NODDI, remain unexplored. Hence, this study aimed to examine the associations between comprehensive lifestyles and *APOE* e4 status—and their potential interactions—with multiple brain structural phenotypes among 24 912 participants without dementia from UK Biobank.

## Materials and methods

### Study design and participants

The UK Biobank is a large community-based cohort with 502 359 participants aged 37–73 years at baseline.^15^ All UK Biobank participants provided written informed consent, and the North West Multi-Centre Ethics Committee granted ethical approval. Since 2014, brain imaging has begun, and this is ongoing until the aim of 100 000 participants being scanned. As of the time of analysis (data last updated September 2023), a total of 71 091 participants aged 44–85 years attended imaging assessment centres for the first time and completed touchscreen questionnaires to provide sociodemographic, physical and medical information. Two-thirds of them had available derived brain MRI phenotypes data. Participants who reported chronic neurological diseases, which could directly affect cognitive function (see Supplementary Table 1) were excluded as previous research did.^14^ This research was conducted using UK Biobank application number 17 689.

### Genetic data

UK Biobank participant genotyping was performed by Affymetrix using a bespoke BiLEVE Axiom array for ∼50 000 individuals and the standard UK Biobank Axiom array for the remaining ∼450 000. All genetic data underwent quality control procedures implemented by UK Biobank.^16^ The *APOE* e genotype is directly genotyped. Further methodological details on genotyping are available from UK Biobank.^17^ For sufficient power to test lifestyle interactions, a dominant e4 model (presence versus absence, namely e3/e4 and e4/e4 collated versus e2/e2, e2/e3 and e3/e3; e2/e4 is usually removed because it has potentially risk and protective alleles)^18^ was used based on previous research because about 2% of the cohort were e4 homozygotes.^14^ *APOE* e4 presence (verses non e4 alleles as absence) was derived based on two directly genotyped single nucleotide polymorphisms (rs7412 and rs429358).

### Lifestyle factors and score

Information on lifestyle variables were derived from questionnaire responses (Supplementary Table 2), including smoking, alcohol drinking, diet, physical activity and sedentary behaviour. The intercorrelations among five lifestyle factors are shown in Supplementary Table 3.

Three-category of each lifestyle factor was used based on guidelines. Smoking was classified as never, forme, and current smoker based on self-reporting as previous study did.^4^ Alcohol drinking was reported as types of alcohol with corresponding units and frequencies per week, and grouped based on national guidelines: 0–14 units average weekly alcohol units as low risk of diseases; 15–35 units for females or 15–50 units for males as moderate risk; > 35 units for females and > 50 units for males as high risk.^19^ Diet was categorized as healthy, moderate, and unhealthy based on a diet score for cardiovascular diseases sharing similar risk factors with dementia rather than the MIND diet,^20^ because data on 24-h diet recalls being used to construct the MIND diet score was not collected when attending the first time MRI assessment. This diet score was from the food frequency questionnaire, and constructed by the unweighted sum of adherence to the UK dietary guideline, including processed meat, red meat individually less than twice per week, total fish more than twice per week, consumption of semi- and skimmed, soya milk, no spread intake, more than five bowls of cereal intake, no salt added to food, more than six glasses of water and more than five servings per day of fruits and vegetables. Sedentary behaviour duration and metabolic equivalents task units (METs) for physical activity were classified as low, medium and high levels according to tertiles.^21^ Tertile was used as there is currently no guideline for sedentary behaviour, and the WHO guideline for physical activity would result in a highly skewed distribution.

Lifestyle score was summed by three-category lifestyle factors using an unweighted score. Two points were assigned for membership of each respective healthy category: never smoking; low risk drinking; healthy diet; low duration sedentariness and high level physical activity. For example, someone who never smoked and had low risk drinking would receive four points. One point was assigned for each of moderate-category lifestyles, such as former smoking, moderate risk drinking, moderate diet, medium duration sedentariness, and medium level physical activity and 0 for unhealthy ones. The lifestyle score ranged from 0 to 10, indicating a healthier lifestyle with a higher score. Based on the distribution of participants with lifestyle scores (see Fig. S1), participants who scored 0, 1, 2 or 3 were classed as unfavourable lifestyle; 4, 5 or 6 as moderate lifestyle; 7, 8, 9 or 10 as favourable lifestyle.

### Imaging data

The brain phenotypes included headsize-uncorrected volumetric makers and WM tracts: grey matter volume, white matter volume, total brain volume, white matter hyperintensity volume (WMHV), left hippocampal volume, right hippocampal volume, (summed to generate) total hippocampal volume, hippocampus volumetric asymmetry (left hippocampal volume minus right),^22^ volumes of frontal lobe GM; FA, MD, intracellular volume fraction (ICVF), isotropic volume fraction (ISOVF) and orientation dispersion (OD).

In this study, all brain structural markers were acquired using brain MRI scans conducted utilizing identical 3T Siemens Skyra scanners. The methodology adhered to a publicly accessible protocol (http://www.fmrib.ox.ac.uk/ukbiobank/protocol/V4_23092014.pdf), supplementary documentation (https://biobank.ctsu.ox.ac.uk/crystal/crystal/docs/brain_mri.pdf).^23^ T1-weighted imaging employed a 3D magnetization-prepared rapid gradient-echo sequence. Initial segmentation of GM and WM was performed via FAST (Automated Segmentation Tool in FMRIB, version 4.1). Subcortical structures, including the hippocampus, were delineated using FIRST (Integrated Registration and Segmentation Tool in FMRIB, version 5.0). WMHV was calculated as the proportion of total white matter volume classified as WMH using T1-weighted and FLAIR MRI data analysed by the BIANCA algorithm.^24^ WMHVs were log-transformed here due to a positively skewed distribution. Global tissue volumes and diffusion metrics from WM tracts, processed and quality-controlled by UK Biobank, were obtained from image-derived phenotypes. Comprehensive details on imaging protocols and quality assurance are outlined in an open-access publication.^23^ Structural MRI outcomes included volumetric measures, while diffusion MRI outcomes comprised NODDI parameters. UK Biobank restricted the image-derived phenotypes to three NODDI metrics: ISOVF, ICVF and OD across WM tracts.

When assessing tractography data, FA and MD are conventional indices of WM tract integrity. FA quantifies the directional coherence of water diffusion, while MD reflects its overall magnitude. Restricted diffusion—caused by dense fibres (e.g. myelinated axons), cell membranes and intracellular structures—elevates FA and reduces MD. Consequently, diminished FA and elevated MD are widely interpreted as markers of diminished white matter integrity.^25^ NODDI provides estimates of neurite density, diffusion of water molecules in the free-water component and neurite orientation dispersion using ICVF, ISOVF and OD, respectively.^26^ Better brain health is anticipated to have a negative correlation with both ISOVF and OD, and a positive correlation with ICVF, which can reflect the dendritic microstructural arrangement and the composition of GM.^27^

We used 27 white matter tracts variables (described in Supplementary Table 4) to construct general factors: FA (gFA), MD (gMD), ICVF (gICVF), ISOVF (gISOVF) and OD (gOD) and general volumes of frontal lobe GM (gFrontal) by principal components analysis.^26^ These general measures demonstrate significant commonality in microstructural characteristics across brain’s major WM tracts, a finding consistent within the present cohort and replicated across diverse populations.^26,28^ The six general components were gFA (eigenvalue = 12.3, 44.9% variance explained), gMD (eigenvalue = 13.4, 49.8% variance explained), gICVF (eigenvalue = 18.6, 68.9% variance explained), gISOVF (eigenvalue = 10.1, 37.4% variance explained), gOD (eigenvalue = 7.3, 26.9% variance explained) and gFrontal (eigenvalue = 6.9, 43.3% variance explained). All brain MRI metrics, initially in raw form, were normalized into *Z*-scores based on the final analysis sample to facilitate interpretation (i.e. similar to standardized betas on a per-SD scale).

### Covariates

Covariates were collected when participants attended their first MRI assessment. Demographics were age at the first scanning and sex. Body mass index (BMI) was grouped as underweight (<18.5 kg/m^2^), normal weight (18.5–24.9 kg/m^2^), overweight (25.0–29.9 kg/m^2^) and obesity (≥30 kg/m^2^) according to WHO guideline.^29^ Sleep duration was assessed with the question ‘About how many hours of sleep do you get in every 24 h?’ and then grouped into three groups: short (<7 h), normal (7–9 h) and long sleep (>9 h) because of potential non-linear association.^30^ Socially isolated status was categorized as ‘yes’ or ‘no’ by assessing one functional and two structural component measures of social connections.^31^ Townsend deprivation score was derived from the postcode of residence and categorized as quintiles.^32^ Higher scores indicated higher levels of area-based socioeconomic deprivation. Participants were asked whether they were diagnosed with each of diabetes, hypertension or depression. Genetic arrays and 10 genetic principal components for stratification were also selected. Intracranial volume (ICV) was generated by summing the volume of WM, GM and ventricular cerebrospinal fluid.^33^

### Statistical analysis

Characteristics were summarized across the *APOE* e4 status (presence versus absence) with the Chi-square tests for categorical variables, and *t*-tests for normally distributed continuous variables.

Linear regression analyses were performed to examine the associations between lifestyle, *APOE* e4 genotype and brain structural phenotypes. First, we examined the associations of lifestyle levels (three-level including unfavourable, moderate and favourable levels), continuous lifestyle score, respectively, with brain structural metrics. We then investigated the associations between *APOE* e4 presence versus absence and brain MRI structural markers. To investigate the unique roles of each lifestyle factor on brain MRI markers, we initially ran a separate model where each individual lifestyle factor with three categories was tested versus each brain structural marker. Unique associations between individual lifestyle factors and brain MRI metrics were quantified via simultaneous multiple linear regression, modelling all lifestyle factors together for each neuroimaging metric of interest. Finally, interactions between *APOE* e4 status with lifestyle levels, and each individual lifestyle aspect on brain structural phenotypes were also tested. If interactions were significant, subgroup analyses were performed. Standardized βs (i.e. on a per-standard deviation scale of effect) and 95% confidence intervals (CIs) were reported.

Two adjustment models were performed to explore whether different confounders impacted the associations. The first model was partially adjusted for age, sex, genotypic array, MRI assessment centre and 10 principal components; ICV was additionally included for volumetric variables including gFrontal. The second model was fully adjusted: additionally, Townsend deprivation score, BMI, sleep duration, isolated status, history of diabetes, hypertension and depression.

All statistical analyses were performed using Stata 18.0 and R (version 4.2.3). We reported two-sided *P-*values throughout, and false discovery rate (FDR) correction *p*_(FDR)_ was applied to keep the error rate at 0.05.

## Results

### Characteristics of participants

A total of 24 912 participants had complete lifestyle factors, genetics, brain imaging markers and covariates at their first MRI assessment (see Supplementary Fig. 2). Descriptive statistics are shown in Table 1. The mean (SD) age was 64.18 (7.62) years. There were 12 368 (49.64%) female participants. One-quarter carried at least one *APOE* e4 allele. Half of the participants had moderate lifestyle, followed by favourable lifestyle (*n =* 10 977/44.06%) and unfavourable lifestyle (*n* *=* 1623/6.51%). The mean (SD) lifestyle score was 6.17 (1.71) points. *APOE* e4 carriers were more likely to have low sedentariness, more physical activity, higher/healthier lifestyle scores and larger grey matter volume compared with non-carriers.

**Table 1:** Characteristics of participants (*N* = 24 912)

	*APOE* e4 absence (*n* = 18 426)	*APOE* e4 presence (*n* = 6486)	*P* values
	*n* (%)	*n* (%)	
Age in years [mean (SD)]	**64.32** (**7.63)**	**63.78** (**7.56)**	<0.001*
Sex			0.313
Female	9113 (49.46)	3255 (50.19)	
Male	9313 (50.54)	3231 (49.81)	
BMI			0.090
Underweight	121 (0.66)	62 (0.96)	
Normal weight	7492 (40.66)	2665 (41.09)	
Obesity	7683 (41.7)	2672 (41.2)	
Overweight	3130 (16.99)	1087 (16.76)	
Sleep duration			0.125
Short sleep	4168 (22.62)	1543 (23.79)	
Normal sleep	14 030 (76.14)	4857 (74.88)	
Long sleep	228 (1.24)	86 (1.33)	
Social-isolated (yes)	1492 (8.1)	500 (7.71)	0.321
Deprivation quintile			0.160
Q1 (least deprived)	3664 (19.88)	1321 (20.37)	
Q2	3712 (20.15)	1274 (19.64)	
Q3	3738 (20.29)	1239 (19.1)	
Q4	3668 (19.91)	1314 (20.26)	
Q5 (most deprived)	3644 (19.78)	1338 (20.63)	
Diabetes (yes)	**1029** (**5.58)**	**308** (**4.75)**	**0.010**
Hypertension (yes)	6026 (32.70)	2057 (31.71)	0.143
Depression (yes)	2277 (12.36)	808 (12.46)	0.833
Smoking			0.085
Current smoker	540 (2.93)	220 (3.39)	
Former smoker	6343 (34.42)	2276 (35.09)	
Never	11 543 (62.65)	3990 (61.52)	
Alcohol drinking			0.556
High risk	966 (5.24)	318 (4.90)	
Moderate risk	6497 (35.26)	2304 (35.52)	
Low risk	10 963 (59.50)	3864 (59.57)	
Diet pattern			0.140
Unhealthy diet	3590 (19.48)	1219 (18.79)	
Moderate diet	12 695 (68.9)	4460 (68.76)	
Healthy diet	2141 (11.62)	807 (12.44)	
Sedentariness			0.032*
High duration	**4781** (**25.95)**	**1610** (**24.82)**	
Medium duration	**7080** (**38.42)**	**2452** (**37.8)**	
Low duration	**6565** (**35.63)**	**2424** (**37.37)**	
Physical activity			0.001*
Low activity	**6256** (**33.95)**	**2048** (**31.58)**	
Medium activity	**6128** (**33.26)**	**2181** (**33.63)**	
High activity	**6042** (**32.79)**	**2257** (**34.8)**	
Lifestyle categories			0.061
Unfavourable	1213 (6.58)	410 (6.32)	
Moderate	9175 (49.79)	3137 (48.37)	
Favourable	8038 (43.62)	2939 (45.31)	
Lifestyle score [mean (SD)]	**6.15** (**1.70)**	**6.22** (**1.72)**	0.002*
Brain MRI values [mean (SD)]			
Grey matter volume (mm^3^)	**616 596.40** (**55 023.75)**	**618 382.70** (**55 223.91)**	**0.025**
White matter volume (mm^3^)	547 944.70 (61 273.84)	548 665.80 (61 560.75)	0.416
Total brain volume (mm^3^)	1 164 541.00 (110 318.00)	1 167 049.00 (110 741.50)	0.116
Left hippocampal volume (mm^3^)	3780.54 (486.74)	3771.57 (483.06)	0.201
Right hippocampal volume (mm^3^)	3898.00 (500.24)	3886.40 (508.45)	0.110
Total hippocampus volume (mm^3^)	7678.55 (895.49)	7657.97 (896.41)	0.112
Hippocampal asymmetry (mm^3^)	117.46 (415.21)	114.83 (424.51)	0.662
White matter hyperintensity volume (mm^3^) [median (IQR)]	2878.00 (4307.00)	2844.00 (4330.00)	0.870

Brain MRI volumes are raw volumes (uncorrected for head size); Bold type indicates *P* < 0.05; *indicates FDR significant [*p*_(FDR)_ < 0.05]; *APOE*, *apolipoprotein E*; BMI, body mass index; IQR, interquartile range; MRI, magnetic resonance imaging; SD, standard deviation.

### Associations of lifestyle levels with brain structural phenotypes

**Figure 1:**
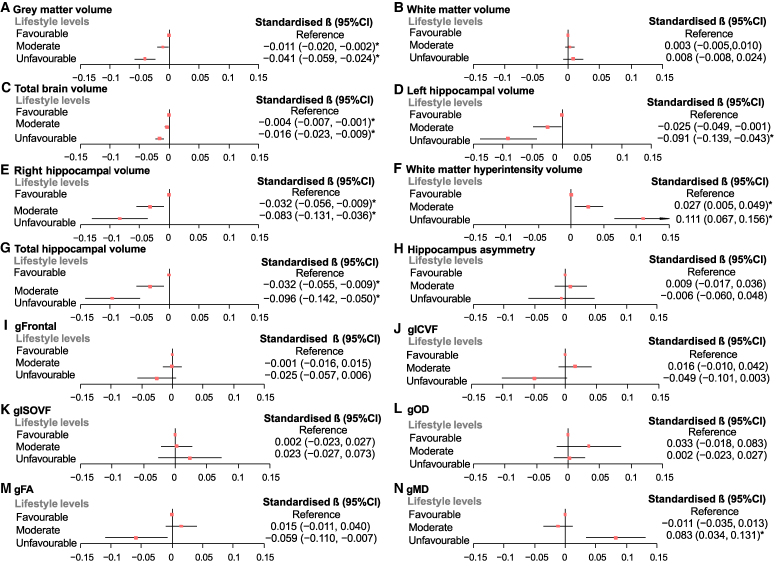
**Forest plots of associations between lifestyle levels and brain structural phenotypes.** Note: standardized βs and 95% confidence intervals (CIs) are reported from regression models where lifestyle factors are regressed onto grey matter volume (**A**), white matter volume (**B**), total brain volume (**C**), left hippocampal volume (**D**), right hippocampal volume (**E**), white matter hyperintensity volume (**F**), total hippocampal volume (**G**), hippocampus asymmetry (**H**), gFrontal (**I**), gICVF (**J**), gISOVF (**K**), gOD (**L**), gFA (**M**) and gMD (**N**) among *N* = 24 912 participants. Fully adjusted: age, sex, genotypic array, four MRI assessment centres, first 10 genetic principal components for general factors; *APOE* e4 status, deprivation index, BMI, sleep duration, isolated status, history of diabetes, hypertension, depression for general factors; ICV additionally adjusted for the volumetric variables. gFrontal, general factors of frontal lobe volumes; gFA, general factors of fractional anisotropy; gICVF, general factors of intracellular volume fraction; gISOVF, general factors of isotropic volume fraction; gMD, general factors of mean diffusivity; gOD, general factors of orientation dispersion. * denotes FDR significant (*p*_(FDR)_ < 0.05).

Forest plots of associations between lifestyle levels and brain imaging phenotypes are reported in Fig. 1. Compared with participants with favourable lifestyles, those with unfavourable and moderate lifestyles were respectively significantly associated with smaller GM, total brain, right and total hippocampal volumes [standardized β range, hereafter simply β (−0.096) to (−0.004), *p*_(FDR)_ < 0.05] and higher log WMHV [β range 0.027 to 0.111, *p*_(FDR)_ < 0.05], indicative of poorer brain health. Of them, those with unfavourable lifestyles were associated with lower values on multiple brain structural markers compared with those with moderate lifestyles (β differences range 0.012–0.084). Moreover, unfavourable lifestyle was associated with poorer white matter tract integrity measures: lower FA [β = −0.059, 95% CI = −0.110 to −0.007, *P* = 0.025, *p*_(FDR)_ = 0.054), higher MD [β = 0.083, 95% CI = 0.034 to 0.131, *P* = 0.001, *p*_(FDR)_ = 0.004] but not significantly associated with more sensitive NODDI measures: ICVF, ISOVF and OD.

### Associations of continuous lifestyle score with brain structural phenotypes

As shown in Table 2, a 1-point higher/healthier lifestyle score was associated with better brain health: larger GM, total brain, left/right hippocampus and total hippocampus [β range 0.002 to 0.014, *p*_(FDR)_ < 0.05] and lower log WMHV [β = −0.017, *p*_(FDR)_ < 0.001] in the fully adjusted models. Notably, 1-point higher was also associated with smaller white matter in the partially adjusted model but became non-significant after full adjustment. All significant associations survived FDR correction in the full adjustment models.

**Table 2:** Associations between continuous lifestyle score and brain structural phenotypes

Brain structural phenotypes	Partially adjusted	95% CI		*P* values	Fully adjusted	95% CI		*P* values
	Standardised β	Lower	Upper		Standardised β	Lower	Upper	
Grey matter volume	**0.011**	**0.009**	**0.013**	<0.001*	**0.006**	**0.003**	**0.008**	<0.001*
White matter volume	**−0.005**	**−0.007**	**−0.003**	<0.001*	−0.001	−0.003	0.001	0.321
Total brain volume	**0.003**	**0.002**	**0.004**	<0.001*	**0.002**	**0.001**	**0.003**	<0.001*
Left hippocampal volume	**0.016**	**0.009**	**0.022**	<0.001*	**0.013**	**0.006**	**0.020**	<0.001*
Right hippocampal volume	**0.013**	**0.006**	**0.019**	<0.001*	**0.012**	**0.005**	**0.019**	0.001*
Whit matter hyperintensity volume	**−0.035**	**−0.041**	**−0.028**	<0.001*	**−0.017**	**−0.024**	**−0.011**	<0.001*
Total hippocampus volume	**0.016**	**0.009**	**0.022**	<0.001*	**0.014**	**0.007**	**0.021**	<0.001*
Hippocampal asymmetry	0.003	−0.005	0.010	0.437	0.001	−0.007	0.009	0.850
gFrontal	0.002	−0.002	0.006	0.403	0.001	−0.003	0.006	0.593
gICVF	−0.002	−0.010	0.005	0.535	0.000	−0.007	0.008	0.911
gISOVF	−0.002	−0.009	0.005	0.546	−0.001	−0.008	0.006	0.847
gOD	−0.006	−0.013	0.001	0.101	−0.003	−0.010	0.004	0.448
gFA	0.003	−0.004	0.011	0.349	0.000	−0.008	0.007	0.898
gMD	−0.005	−0.011	0.002	0.187	−0.005	−0.012	0.002	0.124

Partially adjusted: age, sex, genotypic array, four MRI assessment centres, first 10 genetic principal components for general factors except for gFrontal; ICV additionally adjusted for the volumetric variables; Fully adjusted: additionally, *APOE* e4 status, deprivation index, BMI, sleep duration, isolated status, history of diabetes, hypertension, depression; Bold type indicates *P* < 0.05; *indicates FDR significant [*p*(FDR) < 0.05]; gFA, general factors of fractional anisotropy; gFrontal, general factors of frontal lobe volumes; gICVF, general factors of intracellular volume fraction; gISOVF, general factors of isotropic volume fraction; gMD, general factors of mean diffusivity; gOD, general factors of orientation dispersion.

### Associations between *APOE* e4 status and brain structural phenotypes

As shown in Table 3, there were significant associations between the presence of *APOE* e4 with both hippocampal hemispheres volumes [β range −0.048 to −0.042, *p*_(FDR)_ < 0.05], and log WMHV [β = 0.034, *p*_(FDR)_ = 0.011] in the fully adjusted models. The presence of e4 was significantly associated with worse white matter tract measures: higher ISOVF [β = 0.032, *p*_(FDR)_ = 0.037] and MD [β = 0.040, *p*_(FDR)_ = 0.007], lower FA (β = −0.028, *P* = 0.043) and OD (β = −0.028, *P* = 0.042). The associations between the presence of *APOE* e4 and lower OD and FA did not survive after FDR corrections.

**Table 3:** Associations between APOE e4 status and brain structural phenotypes

Brain structural phenotypes	Partially adjusted	95% CI		*P* values	Fully adjusted	95% CI		*P* values
	Standardised β	Lower	Upper		Standardised β	Lower	Upper	
Grey matter volume	0.000	−0.010	0.009	0.950	−0.001	−0.010	0.008	0.824
White matter volume	0.000	−0.010	0.009	0.950	−0.002	−0.010	0.006	0.618
Total brain volume	−0.001	−0.005	0.002	0.433	−0.002	−0.005	0.002	0.355
Left hippocampus volume	**−0.040**	**−0.065**	**−0.015**	0.002*	**−0.042**	**−0.067**	**−0.016**	0.001*
Right hippocampus volume	**−0.044**	**−0.069**	**−0.019**	0.001*	**−0.045**	**−0.070**	**−0.020**	<0.001*
Whit matter hyperintensity volume	**0.032**	**0.008**	**0.056**	0.008*	**0.034**	**0.011**	**0.058**	0.004*
Total hippocampus volume	**−0.047**	**−0.071**	**−0.022**	<0.001*	**−0.048**	**−0.072**	**−0.023**	<0.001*
Hippocampal asymmetry	0.006	−0.022	0.035	0.663	0.005	−0.023	0.034	0.706
gFrontal	−0.009	−0.026	0.007	0.257	−0.009	−0.026	0.007	0.262
gICVF	−0.026	−0.054	0.001	0.058	−0.026	−0.053	0.002	0.065
gISOVF	**0.033**	**0.006**	**0.059**	0.016*	**0.032**	**0.006**	**0.059**	0.016*
gOD	**−0.028**	**−0.054**	**−0.001**	**0.041**	**−0.028**	**−0.054**	**−0.001**	**0.042**
gFA	**−0.029**	**−0.056**	**−0.001**	**0.039**	**−0.028**	**−0.055**	**−0.001**	**0.043**
gMD	**0.040**	**0.015**	**0.066**	0.002*	**0.040**	**0.014**	**0.065**	0.002*

Partially adjusted: age, sex, genotypic array, four MRI assessment centres, first 10 genetic principal components, for general factors except for gFrontal; ICV additionally adjusted for the volumetric variables; Fully adjusted: additionally, lifestyle levels, deprivation index, BMI, sleep duration, isolated status, history of diabetes, hypertension, depression; Bold type indicates *P* < 0.05; *indicates FDR significant [*p*(FDR) < 0.05]; gFA, general factors of fractional anisotropy; gFrontal, general factors of frontal lobe volumes; gICVF, general factors of intracellular volume fraction; gISOVF, general factors of isotropic volume fraction; gMD, general factors of mean diffusivity; gOD, general factors of orientation dispersion.

### Associations between each lifestyle factor and brain structural phenotypes

**Figure 2:**
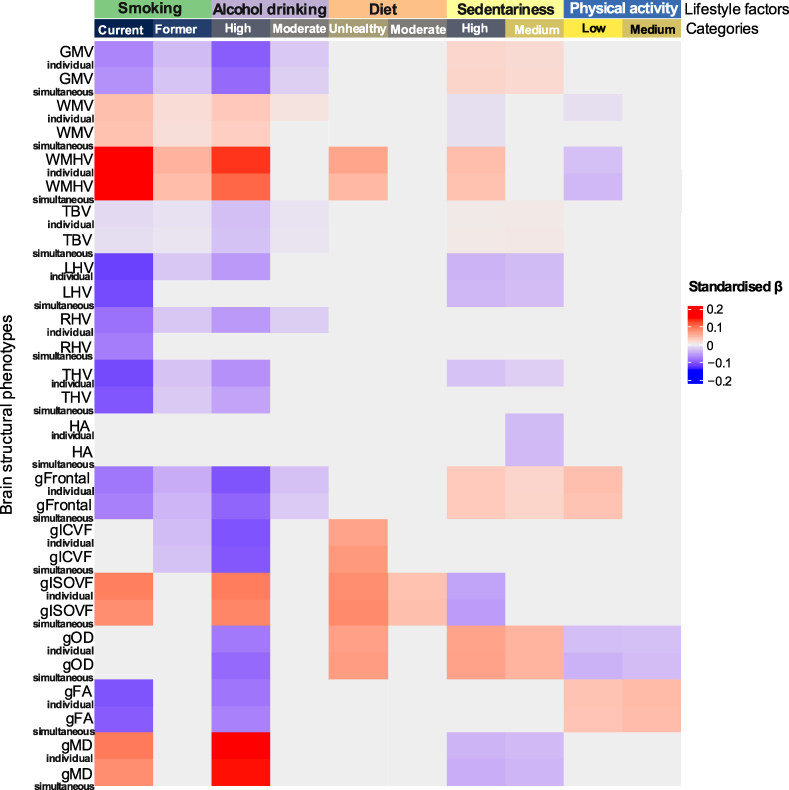
**Heat map of associations between individually and simultaneously modelled lifestyle factors and *APOE* e4 status on brain structural phenotypes.** Note: standardised βs are reported from regression models where each three-level lifestyle factor regressed on brain structural markers individually, then an overall lifestyle factors regressed on brain metrics simultaneously among *N* = 24 912 participants with healthy level-lifestyle as reference, such as, never smoking, low risk of drinking, healthy diet, low duration sedentariness and high level METs. Fully adjusted: age, sex, genotypic array, four MRI assessment centres, first 10 genetic principal components for general factors; *APOE* e4 status, deprivation index, BMI, sleep duration, isolated status, history of diabetes, hypertension, depression for general factors; ICV additionally adjusted for the volumetric variables. Stronger associations are indicated by darker shades, light/dark red indicates a positive association (*P* < 0.05), light/dark purple indicates a negative association (*P* < 0.05). gFA, general factors of fractional anisotropy; gFrontal, general factors of frontal lobe volumes; gICVF, general factors of intracellular volume fraction; gISOVF, general factors of isotropic volume fraction; gMD, general factors of mean diffusivity; GMV, grey matter volume; gOD, general factors of orientation dispersion; HA, hippocampus asymmetry (left minus right); LHV, left hippocampal volume; RHV, right hippocampal volume; TBV, total brain volume; THV, total hippocampus volume; WMHV, white matter hyperintensities volume; WMV, white matter volume.

As shown in Fig. 2, compared with healthy category of each lifestyle factor, unhealthy and moderate categories were generally individually and simultaneously associated with poorer brain health (*P* *<* 0.05). Most significant associations survived from FDR correction [*p*_(FDR)_ *<* 0.05].

Firstly, from the individually modelled lifestyle factor with brain MRI metrics (with supporting information in Table S5), significant associations were generally observed between unhealthy categories of each lifestyle factor and poorer brain health markers. For example, current smoking [β range −0.014 to 0.209, *p*_(FDR)_ < 0.05], and high level drinking [β range −0.105 to 0.152, *p*_(FDR)_ < 0.05], respectively with poorer brain health, across GM and WM, as well as both traditional WM tract and NODDI metrics. Unhealthy diet was associated with worse white matter tract measures: higher log WMHV, ISOVF and OD [β range 0.061–0.079, *p*_(FDR)_ < 0.05]. Interestingly, high sedentariness was observed being associated with better GM health: larger GM, frontal lobe volumes [β range 0.020–0.031, *p*_(FDR)_ < 0.05], but poorer white matter tract measures: higher log WMHV and OD [β range 0.041–0.062, *p*_(FDR)_ < 0.05]. Low levels of physical activity were also associated with better brain health: lower log WMHV, OD and larger general frontal lobe volumes [β range −0.031 to 0.039, *p*_(FDR)_ < 0.05]. Moreover, moderate categories were generally associated with slightly better brain health markers (showing a lighter colour in Fig. 2), compared with those with unhealthy categories (darker colour in Fig. 2). For instance, compared with high drinking level, moderate category was associated with a better brain health, on grey matter volume (*β* *=* *−*0.099 versus −0.026) and frontal lobe volumes (β = −0.104 versus −0.030).

Secondly, in the simultaneous model which included all lifestyle factors together (with supporting information in Table S6), significant associations were also observed between unhealthy and moderate categories of lifestyle and brain structural markers, most of which remained significant as in the individually modelled lifestyle factor with brain MRI metrics. This indicated the contributions of lifestyle factors on brain health were mostly independent, such as current smoking and brain volumes across GM, WM and hippocampus. Yet, some significant associations became non-significant anymore in the simultaneous regression models, indicating that the contributions of certain category of lifestyle factors might be distracted by other lifestyle factors, such as, low level activity and larger hippocampal volumes.

### Interaction of lifestyle levels, each lifestyle factor with *APOE* e4 presence

The interactions between lifestyle levels (i.e. favourable; moderate; unfavourable) and *APOE* e4 status on brain MRI metrics were all non-significant (see Table S7). There were four significant interactions for each lifestyle component by *APOE* e4 presence among alcohol drinking and left and total hippocampus volumes, sedentary behaviour and log WMHV and physical activity and ISOVF shown in Table S8. However, no interactions survived correction for FDR (see Table S8).

## Discussion

This is the first study to examine the potentially interactive associations of overall/individual informative lifestyle factors and *APOE* e4 genotype comprehensively with brain volumes and multiple WM tracts measurements. We found that non-favourable lifestyles were associated with worse brain health across GM, hippocampus and WM tract phenotypes and affirmed that *APOE* e4 presence was associated with smaller hippocampal volumes and worse WM tract integrity. We provide evidence into the relative contributions of each lifestyle factor with brain structural markers: current smoking and high-risk drinking in particular were associated with poorer GM and WM tract measures, and notably, high duration sedentariness was associated with better GM but poorer WM tracts; low level physical activity was associated with better frontal lobe. No interactions were significant after correction for multiple testing with FDR.

### Previous literature

Our results show that people with less healthier lifestyles have on-average worse brain health, consistent with prior studies.^3,13^ A cross-sectional study in UK Biobank showed the relationship of aggregate vascular risk factors (three lifestyle- and four health-based factors) with smaller grey matter volume and larger WMHV, indicating poorer brain health.^13^ Another cross-sectional study of PRECISE cohort also showed that healthier participants adopting four or five healthy lifestyle factors were associated with better brain health: larger GM, total brain and smaller WMH.^3^ Yet, neither study considered the impact of *APOE* e4 genotype and/or multiple informative WM tracts measurements. Compared with these studies, the current report included the *APOE* e4 status as a confounder when investigating the relationship between lifestyle levels and brain health, although the conclusions aligned with previous studies. Moreover, this study investigated 14 brain structural outcomes, including brain volumetric and WM tract variables including aspects of neurite density, which provided comprehensive evidence on the associations modifiable lifestyle factors with certain brain structural markers.

We affirmed significant associations between the carriers of *APOE* e4 and smaller hippocampus among 25k participants considering the environmental impacts of comprehensive lifestyle factors. This finding aligned with previous research showing the influence of the e4 allele on smaller hippocampus volumes.^34^ A positive relationship between *APOE* e4 and WMH was also found, being consistent with previous research in an earlier sub-sample of UK Biobank imaging cohort (*N* *=* 8395).^14^ In a systematic analysis of *APOE* e4 genotype versus blood biomarkers in UK Biobank, Ferguson *et al*.^35^ reported a predominantly cardiometabolic influence of *APOE* e4 genotype on UK Biobank participants without dementia (*N*=∼502k), which aligns with this WMH observation. Our findings reinforce the possibility that *APOE* e4 contributes to age-related cognitive decline partly through a cerebrovascular-type pathway.^36^ NODDI metrics were applied to characterize the relationship of *APOE* e4 on WM microstructure. We found that *APOE* e4 was associated with poorer WM tract integrity phenotypes including FA and MD and ISOVF, indicating less restricted diffusion, representing neuronal loss or inflammation in WM, being consistent with previous research.^37^

All lifestyle factors showed significant associations with some brain structural phenotypes even considering the mutual effects of each other. For example, current smoking, high risk drinking and unhealthy diet were associated with smaller brain volumes/integrity, being consistent with previous research.^4-6^ This might because smoking accelerated brain aging due to atherosclerotic processes i.e. thickening of arteries.^38^ High-level drinking is associated with increased risk of dementia potentially due to the nutritional deficiency, the direct neurotoxic effects of ethanol and the indirect negative impacts through increasing the risk of cardiometabolic diseases (including diabetes, hypertension and stroke).^39^ Unhealthy diet is associated with cardiovascular disease, sharing the same risk factors with cognitive disease.^20,40^

Unexpectedly, high sedentariness and low levels of physical activity were associated with better brain health, conflicting with previous evidence.^41,42^ This could be explained by people with healthier brains may naturally engage in more sedentary activities or occupations, such as cognitive engagement having protective effects on the brain, counteracting the lack of physical activity. Hence, their brain health may allow them to be less physically active without suffering adverse effects. Engaging in physical activity improves cardiorespiratory fitness and cardiovascular heath, which might protect from cognitive decline with age and brain atrophy.^43^ Prolonged sedentary behaviour impairs glucose and lipid metabolism, which are regarded as risk factors for cognitive decline and all-cause dementia.^44^ Further, the upper limit of low levels of MET (1428 MET-min/week) in the current study was much higher than the physical activity recommendations (i.e. ≥ 600 METs),^45^ indicating that participants with medium or high levels of MET might be associated with much better brain health in other studies.

Few studies have explored the relationship between lifestyle factors and NODDI phenotypes. Current smokers showed on average higher ISOVF and MD, and lower FA (i.e. all in the direction of poorer health), being inconsistent with another study reporting null associations.^46^ High-risk drinking was associated with lower ICVF, in line with previous research.^47^ However, the sample sizes in those studies were relatively small (*N* *=* 34–44). We also found that high duration sedentariness was associated with better GM health but poorer WM tracts. This could be explained by vascular and metabolic differences: GM areas might receive adequate blood flow and metabolic support through cognitive activities attributed to high level of sedentary behaviours. This highlights the complex and multifaceted relationship between different types of brain tissue and lifestyle factors, which needs further research.

A few significant interactions of certain categories of lifestyle factors were found. For instance, participants with high risk drinking and the *APOE* e4 carriers with high-risk drinking were associated with larger left hippocampus, being consistent with previous research with a small sample size (*n* *=* 16).^48^ Further research is needed within the larger sample size. There were no statistically significant interactions between lifestyle levels and *APOE* e4 on brain MRI metrics after corrections for FDR, generally aligning with our previous study on cognitive abilities.^49^

### Implications

Our findings suggest that lifestyle interventions may benefit brain health regardless of *APOE* e4 status. Brain health may benefit from a small change towards healthy directions, for multiple modifiable risk factors.^3,14^ Public health strategies promoting healthy habits across the general populations could mitigate neurodegenerative disease risks, while targeted approaches for publicly acknowledged high-risk subgroups (e.g. *APOE* e4 homozygotes and people with unhealthy lifestyles) warrant further investigation, such as smoking cessation, reducing alcohol intake, adopting healthy diets, decreasing sedentary behaviour and maintaining regular physical activity. It is possible to delay the onset of cognitive impairment and dementia, and other neurodegenerative conditions, after modifying the risk factors.

### Limitations

There are some limitations for the current study. First, the cross-sectional study design may limit the possibility of casual inference between lifestyle factors, genetics and brain structures; a longitudinal within-participants design may be more informative. Second, the estimates of associations, particularly pertinent to brain health measures, may be underestimated. Because we have previously observed prevalent healthy bias in the imaging subsample, from the already quite biased UK Biobank baseline sample.^50^ The UK Biobank imaging sample had healthy bias who were relatively more healthy when attending the first MRI assessment. Further research needs to be conducted in more general populations, especially in lower-income countries. Third, the results in our study might be overestimated due to confounding (e.g. disability leads to poorer lifestyle) although we have excluded participants with at least one neurological condition. Fourth, information on lifestyle factors were self-reported, which might influence associations.

## Conclusion

*APOE* e4 and non-favourable lifestyles were consistently associated with poorer brain health after adjustment for confounders. Our findings show that lifestyle has a strong, consistent relationship with multiple aspects of brain health assessed with detailed imaging, and these associations manifest regardless of *APOE* e4 genotypic status. These findings warrant additional large-scale longitudinal studies to confirm and investigate associations across more brain structural phenotypes.

## Supplementary Material

fcaf350_Supplementary_Data

## Data Availability

The data used in this study are available from the UK Biobank. Information regarding registration and application for access is available at https://www.ukbiobank.ac.uk/enable-your-research. The use of data from the UK Biobank was approved by the UK Biobank Access Committee (Project No. 17689). Code for this project is available at https://osf.io/jc6mk/files/osfstorage#.
