# Supplementary material for: Associations and interactions between APOE e4 genotype and lifestyle with brain structural phenotypes
Source: Brain Commun. 2025 Sep 16;7(5):fcaf350. doi: 10.1093/braincomms/fcaf350 (PMC12548027; doi:10.1093/braincomms/fcaf350)
Supplement: fcaf350_Supplementary_Data [file fcaf350_Supplementary_Data.pdf]

Associations and interactions between *APOE* e4 genotype and lifestyle with brain structural phenotypes

Table S1. Self-reported diseases exclusion

Table S2. Lifestyle factors source and definitions

Table S3. Variables included in general factors

Table S4. Lifestyle factors intercorrelations

Table S5. Associations between individual lifestyle factor and brain structural phenotypes

Table S6. Associations between simultaneous lifestyle factors and brain structural phenotypes

Table S7. Individual two-way interactions between APOE e4 genotype and lifestyle levels, on brain structural phenotypes

Table S8. Individual two-way interactions between APOE e4 genotype and each lifestyle factor, on brain structural phenotypes

Figure S1. Histogram showing the distribution of lifestyle scores

Figure S2. Flowchart summarising participants selection and inclusion processes, including the reasons for exclusion of participants

Table S1. Self-reported diseases exclusion

Diseases
Brain cancer/primary malignant tumour
Brain haemorrhage
Brain/intracranial abscess
Cerebral aneurysm
Cerebral palsy
Chronic/degenerative neurological problem
Dementia/Alzheimer's disease/cognitive impairment
Encephalitis
Epilepsy
Head injury
Infection of nervous system
Ischaemic stroke
Meningeal cancer/malignant meningioma
Meningioma (benign)
Meningitis
Motor neurone disease
Multiple sclerosis
Neurological injury/trauma
Neuroma (benign)
Other demyelinating condition
Other neurological problem
Parkinson's disease
Spina bifida
Stroke
Subarachnoid haemorrhage
Subdural haematoma
Transient ischaemic attack
Brain haemorrhage

Table S2. Lifestyle factors source and definitions

Lifestyle factor	Source and definition
Smoking status	UK Biobank Touchscreen questionnaire at MRI assessment. Smoking status defined as current, previous, never smoker.
Alcohol drinking	UK Biobank Touchscreen questionnaire at MRI assessment. Alcohol drinking was calculated by alcohol intake frequency; average weekly red wine intake; average weekly champagne plus white wine intake; average weekly beer plus cider intake; average weekly spirits intake; average weekly fortified wine intake based on UK alcohol units guideline.
Diet	UK Biobank Touchscreen questionnaire at MRI assessment. Diet score was constructed by the unweighted sum of adherence to the UK dietary guideline of processed meat intake; beef intake; lamb intake; pork intake; oily fish; non-oily fish; milk type; spread; cereal; adding salt to food; fresh fruit; dried fruit; salad/raw vegetables; cooked vegetables; water.
Physical activity	UK Biobank Touchscreen questionnaire at MRI assessment. Physical activity was calculated by MET minutes per week for moderate activity; MET minutes per week for vigorous activity; MET minutes per week for walking.
Sedentary behaviour	UK Biobank Touchscreen questionnaire at MRI assessment. Sedentary behaviour was calculated by summing the time spent for driving, time spent for personal computer, and time spent for watching television.

Note: MRI, magnetic resonance imaging; MET, metabolic equivalents task units.

Table S3. Lifestyle factors intercorrelations

Standardised β	Smoking	Alcohol drinking	Diet	Sedentariness	Physical activity
Smoking	1				
Alcohol drinking	**0.1865**	1			
Diet	**0.0698**	**0.1461**	1		
Sedentariness	**0.0591**	**0.0699**	**0.1140**	1	
Physical activity	**0.0139**	**-0.0130**	**0.0922**	**0.0996**	1

Note. Standardised βs reported. Bold type means $p<0.05$

Table S4. Variables included in general factors

Description	Contents
Variables included in "gFrontal"	
	Volume of grey matter in Frontal Pole (left)
	Volume of grey matter in Frontal Pole (right)
	Volume of grey matter in Superior Frontal Gyrus (left)
	Volume of grey matter in Superior Frontal Gyrus (right)
	Volume of grey matter in Middle Frontal Gyrus (left)
	Volume of grey matter in Middle Frontal Gyrus (right)
	Volume of grey matter in Inferior Frontal Gyrus, pars triangularis (left)
	Volume of grey matter in Inferior Frontal Gyrus, pars triangularis (right)
	Volume of grey matter in Inferior Frontal Gyrus, pars opercularis (left)
	Volume of grey matter in Inferior Frontal Gyrus, pars opercularis (right)
	Volume of grey matter in Frontal Medial Cortex (left)
	Volume of grey matter in Frontal Medial Cortex (right)
	Volume of grey matter in Frontal Orbital Cortex (left)
	Volume of grey matter in Frontal Orbital Cortex (right)
	Volume of grey matter in Frontal Operculum Cortex (left)
	Volume of grey matter in Frontal Operculum Cortex (right)
Variables included in "gFA"	
	Weighted-mean FA in tract acoustic radiation (left)
	Weighted-mean FA in tract acoustic radiation (right)
	Weighted-mean FA in tract anterior thalamic radiation (left)
	Weighted-mean FA in tract anterior thalamic radiation (right)
	Weighted-mean FA in tract cingulate gyrus part of cingulum (left)
	Weighted-mean FA in tract cingulate gyrus part of cingulum (right)
	Weighted-mean FA in tract parahippocampal part of cingulum (left)
	Weighted-mean FA in tract parahippocampal part of cingulum (right)
	Weighted-mean FA in tract corticospinal tract (left)
	Weighted-mean FA in tract corticospinal tract (right)
	Weighted-mean FA in tract forceps major
	Weighted-mean FA in tract forceps minor
	Weighted-mean FA in tract inferior fronto-occipital fasciculus (left)
	Weighted-mean FA in tract inferior fronto-occipital fasciculus (right)
	Weighted-mean FA in tract inferior longitudinal fasciculus (left)
	Weighted-mean FA in tract inferior longitudinal fasciculus (right)
	Weighted-mean FA in tract middle cerebellar peduncle
	Weighted-mean FA in tract medial lemniscus (left)
	Weighted-mean FA in tract medial lemniscus (right)
	Weighted-mean FA in tract posterior thalamic radiation (left)
	Weighted-mean FA in tract posterior thalamic radiation (right)
	Weighted-mean FA in tract superior longitudinal fasciculus (left)
	Weighted-mean FA in tract superior longitudinal fasciculus (right)
	Weighted-mean FA in tract superior thalamic radiation (left)
	Weighted-mean FA in tract superior thalamic radiation (right)
	Weighted-mean FA in tract uncinate fasciculus (left)
	Weighted-mean FA in tract uncinate fasciculus (right)
Variables included in "gMD"	
	Weighted-mean MD in tract acoustic radiation (left)
	Weighted-mean MD in tract acoustic radiation (right)
	Weighted-mean MD in tract anterior thalamic radiation (left)
	Weighted-mean MD in tract anterior thalamic radiation (right)
	Weighted-mean MD in tract cingulate gyrus part of cingulum (left)
	Weighted-mean MD in tract cingulate gyrus part of cingulum (right)
	Weighted-mean MD in tract parahippocampal part of cingulum (left)
	Weighted-mean MD in tract parahippocampal part of cingulum (right)
	Weighted-mean MD in tract corticospinal tract (left)
	Weighted-mean MD in tract corticospinal tract (right)
	Weighted-mean MD in tract forceps major
	Weighted-mean MD in tract forceps minor
	Weighted-mean MD in tract inferior fronto-occipital fasciculus (left)
	Weighted-mean MD in tract inferior fronto-occipital fasciculus (right)
	Weighted-mean MD in tract inferior longitudinal fasciculus (left)
	Weighted-mean MD in tract inferior longitudinal fasciculus (right)
	Weighted-mean MD in tract middle cerebellar peduncle
	Weighted-mean MD in tract medial lemniscus (left)
	Weighted-mean MD in tract medial lemniscus (right)
	Weighted-mean MD in tract posterior thalamic radiation (left)
	Weighted-mean MD in tract posterior thalamic radiation (right)
	Weighted-mean MD in tract superior longitudinal fasciculus (left)
	Weighted-mean MD in tract superior longitudinal fasciculus (right)
	Weighted-mean MD in tract superior thalamic radiation (left)
	Weighted-mean MD in tract superior thalamic radiation (right)

	Weighted-mean MD in tract uncinate fasciculus (left)
	Weighted-mean MD in tract uncinate fasciculus (right)
Variables included in "gICVF"	
	Weighted-mean ICVF in tract acoustic radiation (left)
	Weighted-mean ICVF in tract acoustic radiation (right)
	Weighted-mean ICVF in tract anterior thalamic radiation (left)
	Weighted-mean ICVF in tract anterior thalamic radiation (right)
	Weighted-mean ICVF in tract cingulate gyrus part of cingulum (left)
	Weighted-mean ICVF in tract cingulate gyrus part of cingulum (right)
	Weighted-mean ICVF in tract parahippocampal part of cingulum (left)
	Weighted-mean ICVF in tract parahippocampal part of cingulum (right)
	Weighted-mean ICVF in tract corticospinal tract (left)
	Weighted-mean ICVF in tract corticospinal tract (right)
	Weighted-mean ICVF in tract forceps major
	Weighted-mean ICVF in tract forceps minor
	Weighted-mean ICVF in tract inferior fronto-occipital fasciculus (left)
	Weighted-mean ICVF in tract inferior fronto-occipital fasciculus (right)
	Weighted-mean ICVF in tract inferior longitudinal fasciculus (left)
	Weighted-mean ICVF in tract inferior longitudinal fasciculus (right)
	Weighted-mean ICVF in tract middle cerebellar peduncle
	Weighted-mean ICVF in tract medial lemniscus (left)
	Weighted-mean ICVF in tract medial lemniscus (right)
	Weighted-mean ICVF in tract posterior thalamic radiation (left)
	Weighted-mean ICVF in tract posterior thalamic radiation (right)
	Weighted-mean ICVF in tract superior longitudinal fasciculus (left)
	Weighted-mean ICVF in tract superior longitudinal fasciculus (right)
	Weighted-mean ICVF in tract superior thalamic radiation (left)
	Weighted-mean ICVF in tract superior thalamic radiation (right)
	Weighted-mean ICVF in tract uncinate fasciculus (left)
	Weighted-mean ICVF in tract uncinate fasciculus (right)
Variables included in "gISOVF"	
	Weighted-mean ISOVF in tract acoustic radiation (left)
	Weighted-mean ISOVF in tract acoustic radiation (right)
	Weighted-mean ISOVF in tract anterior thalamic radiation (left)
	Weighted-mean ISOVF in tract anterior thalamic radiation (right)
	Weighted-mean ISOVF in tract cingulate gyrus part of cingulum (left)
	Weighted-mean ISOVF in tract cingulate gyrus part of cingulum (right)
	Weighted-mean ISOVF in tract parahippocampal part of cingulum (left)
	Weighted-mean ISOVF in tract parahippocampal part of cingulum (right)
	Weighted-mean ISOVF in tract corticospinal tract (left)
	Weighted-mean ISOVF in tract corticospinal tract (right)
	Weighted-mean ISOVF in tract forceps major
	Weighted-mean ISOVF in tract forceps minor
	Weighted-mean ISOVF in tract inferior fronto-occipital fasciculus (left)
	Weighted-mean ISOVF in tract inferior fronto-occipital fasciculus (right)
	Weighted-mean ISOVF in tract inferior longitudinal fasciculus (left)
	Weighted-mean ISOVF in tract inferior longitudinal fasciculus (right)
	Weighted-mean ISOVF in tract middle cerebellar peduncle
	Weighted-mean ISOVF in tract medial lemniscus (left)
	Weighted-mean ISOVF in tract medial lemniscus (right)
	Weighted-mean ISOVF in tract posterior thalamic radiation (left)
	Weighted-mean ISOVF in tract posterior thalamic radiation (right)
	Weighted-mean ISOVF in tract superior longitudinal fasciculus (left)
	Weighted-mean ISOVF in tract superior longitudinal fasciculus (right)
	Weighted-mean ISOVF in tract superior thalamic radiation (left)
	Weighted-mean ISOVF in tract superior thalamic radiation (right)
	Weighted-mean ISOVF in tract uncinate fasciculus (left)
	Weighted-mean ISOVF in tract uncinate fasciculus (right)
Variables included in "gOD"	
	Weighted-mean OD in tract acoustic radiation (left)
	Weighted-mean OD in tract acoustic radiation (right)
	Weighted-mean OD in tract anterior thalamic radiation (left)
	Weighted-mean OD in tract anterior thalamic radiation (right)
	Weighted-mean OD in tract cingulate gyrus part of cingulum (left)
	Weighted-mean OD in tract cingulate gyrus part of cingulum (right)
	Weighted-mean OD in tract parahippocampal part of cingulum (left)
	Weighted-mean OD in tract parahippocampal part of cingulum (right)
	Weighted-mean OD in tract corticospinal tract (left)
	Weighted-mean OD in tract corticospinal tract (right)
	Weighted-mean OD in tract forceps major
	Weighted-mean OD in tract forceps minor
	Weighted-mean OD in tract inferior fronto-occipital fasciculus (left)
	Weighted-mean OD in tract inferior fronto-occipital fasciculus (right)
	Weighted-mean OD in tract inferior longitudinal fasciculus (left)
	Weighted-mean OD in tract inferior longitudinal fasciculus (right)

	Weighted-mean OD in tract middle cerebellar peduncle
	Weighted-mean OD in tract medial lemniscus (left)
	Weighted-mean OD in tract medial lemniscus (right)
	Weighted-mean OD in tract posterior thalamic radiation (left)
	Weighted-mean OD in tract posterior thalamic radiation (right)
	Weighted-mean OD in tract superior longitudinal fasciculus (left)
	Weighted-mean OD in tract superior longitudinal fasciculus (right)
	Weighted-mean OD in tract superior thalamic radiation (left)
	Weighted-mean OD in tract superior thalamic radiation (right)
	Weighted-mean OD in tract uncinate fasciculus (left)
	Weighted-mean OD in tract uncinate fasciculus (right)

Note: gFrontal, general factors of frontal lobe volumes; gICVF, general factors of intracellular volume fraction; gISOVF, general factors of isotropic volume fraction; gOD, general factors of orientation dispersion; gFA, general factors of fractional anisotropy; gMD, general factors of mean diffusivity.

Table S5. Associations between individual lifestyle factor and brain structural phenotypes

Standardised β (95% CI) / Brain structural phenotypes	Smoking		Alcohol drinking		Diet		Sedentariness		Physical activity	
	Current	Former	High	Moderate	Unhealthy	Moderate	High	Medium	Low	Medium
GMV	**-0.071*<br>(-0.095, -0.048)**	**-0.035*<br>(-0.043, -0.026)**	**-0.099 *<br>(-0.118, -0.080)**	**-0.026 *<br>(-0.035, -0.016)**	-0.004<br>(-0.019, 0.012)	-0.001<br>(-0.014, 0.012)	**0.020 *<br>(0.009, 0.031)**	**0.017 *<br>(0.007, 0.026)**	0.008<br>(-0.002, 0.018)	0.003<br>(-0.007, 0.012)
WMV	**0.040 *<br>(0.018, 0.061)**	**0.015*<br>(0.007, 0.023)**	**0.032 *<br>(0.015, 0.049)**	**0.010<br>(0.001, 0.018)**	0.001<br>(-0.013, 0.015)	0.002<br>(-0.010, 0.013)	**-0.010<br>(-0.020, 0.000)**	-0.006<br>(-0.014, 0.003)	**-0.010<br>(-0.019, -0.001)**	0.001<br>(-0.008, 0.010)
TBV	**-0.014*<br>(-0.023, -0.004)**	**-0.009*<br>(-0.012, -0.005)**	**-0.032 *<br>(-0.039, -0.024)**	**-0.007 *<br>(-0.011, -0.004)**	-0.001<br>(-0.007, 0.005)	0.001<br>(-0.004, 0.006)	**0.004<br>(0.000, 0.009)**	**0.005 *<br>(0.001, 0.009)**	-0.001<br>(-0.005, 0.002)	0.002<br>(-0.002, 0.006)
LHV	**-0.117*<br>(-0.182, -0.052)**	**-0.026*<br>(-0.050, -0.003)**	**-0.058<br>(-0.110, -0.006)**	-0.005<br>(-0.030, 0.019)	-0.011<br>(-0.053, 0.031)	0.010<br>(-0.025, 0.045)	**-0.040 *<br>(-0.070, -0.010)**	**-0.034 *<br>(-0.061, -0.008)**	-0.011<br>(-0.038, 0.017)	-0.002<br>(-0.029, 0.025)
RHV	**-0.085*<br>(-0.150, -0.021)**	**-0.027<br>(-0.050, -0.003)**	**-0.058<br>(-0.109, -0.006)**	-0.022<br>(-0.046, 0.003)	-0.017<br>(-0.058, 0.025)	0.000<br>(-0.035, 0.034)	-0.014<br>(-0.043, 0.016)	-0.005<br>(-0.030, 0.021)	-0.021<br>(-0.049, 0.006)	0.010<br>(-0.017, 0.037)
WMHV	**0.209*<br>(0.148, 0.270)**	**0.049*<br>(0.027, 0.072)**	**0.133 *<br>(0.085, 0.182)**	0.023<br>(0.000, 0.046)	**0.061 *<br>(0.022, 0.099)**	0.024<br>(-0.009, 0.056)	**0.041 *<br>(0.013, 0.069)**	0.011<br>(-0.013, 0.035)	**-0.031 *<br>(-0.057, -0.006)**	-0.023<br>(-0.048, 0.002)
THV	**-0.111*<br>(-0.174, -0.048)**	**-0.029*<br>(-0.052, -0.006)**	**-0.064<br>(-0.114, -0.014)**	-0.015<br>(-0.039, 0.009)	-0.015<br>(-0.055, 0.025)	0.006<br>(-0.028, 0.039)	**-0.029<br>(-0.058, -0.001)**	-0.021<br>(-0.046, 0.004)	-0.018<br>(-0.044, 0.009)	0.005<br>(-0.022, 0.031)
HA	-0.033<br>(-0.106, 0.040)	0.001<br>(-0.025, 0.028)	0.002<br>(-0.056, 0.060)	0.020<br>(-0.008, 0.048)	0.007<br>(-0.039, 0.054)	0.013<br>(-0.027, 0.052)	-0.030<br>(-0.063, 0.004)	**-0.035<br>(-0.064, -0.005)**	0.013<br>(-0.018, 0.044)	-0.014<br>(-0.045, 0.016)
gFrontal	**-0.081*<br>(-0.124, -0.039)**	**-0.044*<br>(-0.059, -0.028)**	**-0.104 *<br>(-0.138, -0.071)**	**-0.030 *<br>(-0.046, -0.014)**	0.005<br>(-0.022, 0.032)	0.000<br>(-0.022, 0.023)	**0.031 *<br>(0.012, 0.051)**	**0.021 *<br>(0.004, 0.038)**	**0.039 *<br>(0.021, 0.057)**	0.011<br>(-0.007, 0.028)
gICVF	-0.069<br>(-0.140, 0.001)	**-0.034*<br>(-0.060, -0.008)**	**-0.105 *<br>(-0.161, -0.049)**	-0.014<br>(-0.041, 0.013)	**0.062 *<br>(0.017, 0.107)**	0.036<br>(-0.002, 0.074)	0.026<br>(-0.006, 0.058)	0.017<br>(-0.011, 0.045)	0.009<br>(-0.021, 0.039)	0.021<br>(-0.008, 0.051)
gISOVF	**0.090*<br>(0.022, 0.158)**	-0.007<br>(-0.031, 0.018)	**0.092 *<br>(0.038, 0.146)**	-0.004<br>(-0.030, 0.021)	**0.079 *<br>(0.036, 0.123)**	**0.037<br>(0.001, 0.074)**	**-0.051 *<br>(-0.082, -0.020)**	-0.025<br>(-0.052, 0.003)	-0.015<br>(-0.044, 0.014)	-0.018<br>(-0.046, 0.010)
gOD	0.025<br>(-0.044, 0.094)	-0.019<br>(-0.044, 0.006)	**-0.080 *<br>(-0.134, -0.025)**	-0.013<br>(-0.039, 0.013)	**0.065 *<br>(0.021, 0.108)**	0.013<br>(-0.024, 0.050)	**0.062 *<br>(0.031, 0.094)**	**0.049 *<br>(0.021, 0.076)**	**-0.033<br>(-0.062, -0.004)**	**-0.031<br>(-0.059, -0.002)**
gFA	**-0.104*<br>(-0.174, -0.035)**	-0.013<br>(-0.038, 0.013)	**-0.082 *<br>(-0.138, -0.027)**	0.007<br>(-0.019, 0.034)	0.018<br>(-0.027, 0.062)	0.036<br>(-0.001, 0.074)	0.004<br>(-0.028, 0.036)	-0.010<br>(-0.038, 0.018)	**0.036 *<br>(0.007, 0.065)**	**0.043 *<br>(0.014, 0.072)**
gMD	**0.094*<br>(0.027, 0.160)**	0.022<br>(-0.002, 0.046)	**0.152 *<br>(0.100, 0.204)**	0.023<br>(-0.002, 0.048)	0.035<br>(-0.007, 0.077)	0.015<br>(-0.021, 0.050)	**-0.039 *<br>(-0.069, -0.009)**	**-0.036 *<br>(-0.062, -0.009)**	-0.011<br>(-0.039, 0.017)	-0.019<br>(-0.046, 0.009)

Note: Standardised betas (β) and 95% confidence intervals are reported from regression models where each risk factors are individually regressed onto brain phenotypes in the fully adjusted models with the healthiest level as reference, such as, never smoking, low risk of drinking, healthy diet, low duration sedentariness, high level MET. Betas reflect increases per SD of the dependent variable, per increase in the independent variable. Bold type indicates P<0.05; * indicates FDR significant ($p_{(FDR)}<0.05$). CI, confidence interval; GMV, grey matter volume; WMV, white matter volume; TBV, total brain volume; LHV, left hippocampal volume; RHV, right hippocampal volume; WMHV, white matter hyperintensities volume; THV, total hippocampus volume; HA, hippocampus asymmetry (left hippocampal volume minus right); gFrontal, general factors of frontal lobe volumes; gICVF, general factors of intracellular volume fraction; gISOVF, general factors of isotropic volume fraction; gOD, general factors of orientation dispersion; gFA, general factors of fractional anisotropy; gMD, general factors of mean diffusivity.

Table S6. Associations between simultaneous lifestyle factors and brain structural phenotypes

Standardised β (95% CI)	Smoking		Alcohol drinking		Diet		Sedentariness		Physical activity	
Brain structural phenotypes	Current	Former	High	Moderate	Unhealthy	Moderate	High	Medium	Low	Medium
GMV	**-0.063*<br>(-0.087, -0.040)**	**-0.029 *<br>(-0.037, -0.020)**	**-0.090 *<br>(-0.109, -0.071)**	**-0.021*<br>(-0.030, -0.012)**	0.004<br>(-0.012, 0.019)	0.001<br>(-0.012, 0.014)	**0.022 *<br>(0.011, 0.033)**	**0.018 *<br>(0.008, 0.027)**	0.006<br>(-0.004, 0.016)	0.001<br>(-0.009, 0.011)
WMV	**0.038*<br>(0.016, 0.059)**	**0.013 *<br>(0.005, 0.021)**	**0.027 *<br>(0.010, 0.045)**	0.007<br>(-0.001, 0.015)	-0.001<br>(-0.015, 0.013)	0.001<br>(-0.010, 0.013)	-0.010<br>(-0.020, 0.000)	-0.006<br>(-0.015, 0.003)	-0.009<br>(-0.018, 0.000)	0.002<br>(-0.007, 0.011)
TBV	**-0.011<br>(-0.020, -0.001)**	**-0.007 *<br>(-0.010, -0.003)**	**-0.030 *<br>(-0.037, -0.022)**	**-0.007 *<br>(-0.010, -0.003)**	0.001<br>(-0.005, 0.007)	0.001<br>(-0.004, 0.006)	**0.005 *<br>(0.001, 0.010)**	**0.006 *<br>(0.002, 0.009)**	-0.002<br>(-0.006, 0.002)	0.001<br>(-0.002, 0.005)
LHV	**-0.111 *<br>(-0.176, -0.045)**	-0.023<br>(-0.047, 0.001)	-0.045<br>(-0.097, 0.008)	0.000<br>(-0.025, 0.025)	0.001<br>(-0.041, 0.043)	0.015<br>(-0.020, 0.050)	**-0.037 *<br>(-0.067, -0.007)**	**-0.034 *<br>(-0.060, -0.007)**	-0.008<br>(-0.035, 0.020)	-0.002<br>(-0.029, 0.025)
RHV	**-0.077<br>(-0.142, -0.012)**	-0.022<br>(-0.046, 0.002)	-0.047<br>(-0.099, 0.005)	-0.018<br>(-0.043, 0.007)	-0.005<br>(-0.047, 0.037)	0.004<br>(-0.031, 0.039)	-0.009<br>(-0.039, 0.020)	-0.003<br>(-0.029, 0.023)	-0.021<br>(-0.048, 0.007)	0.010<br>(-0.017, 0.036)
WMHV	**0.194 *<br>(0.133, 0.255)**	**0.041 *<br>(0.019, 0.064)**	**0.107 *<br>(0.058, 0.155)**	0.011<br>(-0.012, 0.035)	**0.045<br>(0.006, 0.085)**	0.019<br>(-0.014, 0.052)	**0.037 *<br>(0.009, 0.065)**	0.009<br>(-0.015, 0.033)	**-0.036 *<br>(-0.062, -0.010)**	-0.023<br>(-0.048, 0.003)
THV	**-0.103 *<br>(-0.167, -0.040)**	**-0.025<br>(-0.048, -0.002)**	**-0.051<br>(-0.101, 0.000)**	-0.010<br>(-0.034, 0.014)	-0.002<br>(-0.043, 0.038)	0.010<br>(-0.024, 0.044)	-0.025<br>(-0.054, 0.004)	-0.020<br>(-0.045, 0.005)	-0.016<br>(-0.043, 0.011)	0.004<br>(-0.022, 0.030)
HA	-0.036<br>(-0.110, 0.038)	-0.001<br>(-0.028, 0.026)	0.005<br>(-0.054, 0.064)	0.022<br>(-0.007, 0.050)	0.007<br>(-0.040, 0.054)	0.012<br>(-0.027, 0.052)	-0.032<br>(-0.065, 0.002)	**-0.036<br>(-0.065, -0.006)**	0.016<br>(-0.015, 0.047)	-0.014<br>(-0.044, 0.017)
gFrontal	**-0.075 *<br>(-0.117, -0.032)**	**-0.038 *<br>(-0.053, -0.022)**	**-0.093 *<br>(-0.127, -0.060)**	**-0.024 *<br>(-0.041, -0.008)**	0.009<br>(-0.018, 0.036)	0.001<br>(-0.022, 0.023)	**0.031 *<br>(0.011, 0.050)**	**0.022 *<br>(0.005, 0.039)**	0.036 *<br>(0.018, 0.054)	0.009<br>(-0.009, 0.026)
gICVF	-0.067<br>(-0.138, 0.004)	**-0.030<br>(-0.056, -0.004)**	**-0.103 *<br>(-0.159, -0.046)**	-0.013<br>(-0.040, 0.015)	**0.070 *<br>(0.025, 0.116)**	0.038<br>(0.000, 0.075)	0.025<br>(-0.007, 0.057)	0.017<br>(-0.012, 0.045)	0.003<br>(-0.026, 0.033)	0.019<br>(-0.011, 0.048)
gISOVF	**0.079<br>(0.011, 0.148)**	-0.010<br>(-0.036, 0.015)	**0.084 *<br>(0.029, 0.138)**	-0.007<br>(-0.034, 0.019)	**0.081 *<br>(0.037, 0.125)**	**0.040<br>(0.003, 0.076)**	**-0.056*<br>(-0.087, -0.025)**	-0.026<br>(-0.054, 0.001)	-0.015<br>(-0.044, 0.014)	-0.017<br>(-0.045, 0.011)
gOD	0.024<br>(-0.045, 0.093)	-0.016<br>(-0.041, 0.009)	**-0.091*<br>(-0.146, -0.035)**	-0.016<br>(-0.043, 0.010)	**0.068 *<br>(0.024, 0.112)**	0.013<br>(-0.024, 0.050)	**0.063 *<br>(0.031, 0.094)**	**0.049 *<br>(0.021, 0.076)**	**-0.041 *<br>(-0.070, -0.012)**	**-0.033<br>(-0.062, -0.005)**
gFA	**-0.100 *<br>(-0.170, -0.030)**	-0.010<br>(-0.036, 0.016)	**-0.074 *<br>(-0.130, -0.018)**	0.011<br>(-0.016, 0.037)	0.021<br>(-0.024, 0.066)	0.036<br>(-0.002, 0.073)	0.003<br>(-0.029, 0.035)	-0.011<br>(-0.039, 0.017)	**0.035<br>(0.006, 0.065)**	**0.041 *<br>(0.012, 0.071)**
gMD	**0.079<br>(0.013, 0.145)**	0.013<br>(-0.011, 0.038)	**0.143 *<br>(0.090, 0.196)**	0.019<br>(-0.006, 0.045)	0.027<br>(-0.015, 0.070)	0.014<br>(-0.022, 0.049)	**-0.044 *<br>(-0.074, -0.013)**	**-0.038 *<br>(-0.064, -0.011)**	-0.009<br>(-0.037, 0.019)	-0.016<br>(-0.044, 0.011)

Note: Standardised betas (β) and 95% confidence intervals are reported from regression models where each risk factors are simultaneous regressed onto brain phenotypes in the fully adjusted models with the healthiest level as reference, such as, never smoking, low risk of drinking, healthy diet, low duration sedentariness, high level MET. Betas reflect increases per SD of the dependent variable, per increase in the independent variable. Bold type indicates $P<0.05$; * indicates FDR significant ($p_{(FDR)} <0.05$). CI, confidence interval; GMV, grey matter volume; WMV, white matter volume; TBV, total brain volume; LHV, left hippocampal volume; RHV, right hippocampal volume; WMHV, white matter hyperintensities volume; THV, total hippocampus volume; HA, hippocampus asymmetry (left hippocampal volume minus right); gFrontal, general factors of frontal lobe volumes; gICVF, general factors of intracellular volume fraction; gISOVF, general factors of isotropic volume fraction; gOD, general factors of orientation dispersion; gFA, general factors of fractional anisotropy; gMD, general factors of mean diffusivity.

Table S7. Individual two-way interactions between *APOE* e4 genotype and lifestyle levels, on brain structural phenotypes

Brain structural phenotypes	Standardised β	95% CI Lower	Upper	P values
**Grey matter volume**				
Present × Unfavourable	-0.005	-0.044	0.034	0.798
Present × Moderate	0.008	-0.011	0.027	0.413
**White matter volume**				
Present × Unfavourable	-0.002	-0.037	0.033	0.926
Present × Moderate	-0.007	-0.024	0.010	0.423
**Total brain volume**				
Present × Unfavourable	-0.003	-0.019	0.012	0.655
Present × Moderate	0.000	-0.007	0.007	0.982
**Left hippocampus volume**				
Present × Unfavourable	0.048	-0.059	0.154	0.379
Present × Moderate	-0.002	-0.054	0.051	0.951
**Right hippocampus volume**				
Present × Unfavourable	0.005	-0.100	0.111	0.921
Present × Moderate	0.024	-0.028	0.076	0.363
**White matter hyperintensity volume**				
Present × Unfavourable	0.002	-0.098	0.101	0.973
Present × Moderate	0.016	-0.033	0.064	0.520
**Total hippocampus volume**				
Present × Unfavourable	0.029	-0.074	0.132	0.581
Present × Moderate	0.013	-0.038	0.063	0.625
**Hippocampus asymmetry**				
Present × Unfavourable	0.049	-0.070	0.169	0.420
Present × Moderate	-0.031	-0.089	0.028	0.304
**gFrontal**				
Present × Unfavourable	-0.001	-0.070	0.068	0.984
Present × Moderate	-0.003	-0.037	0.031	0.850
**gICVF**				
Present × Unfavourable	-0.025	-0.140	0.091	0.676
Present × Moderate	0.042	-0.015	0.098	0.147
**gISOVF**				
Present × Unfavourable	0.054	-0.057	0.166	0.337
Present × Moderate	0.043	-0.011	0.098	0.119
**gOD**				
Present × Unfavourable	-0.038	-0.150	0.075	0.510
Present × Moderate	0.018	-0.037	0.073	0.519
**gFA**				
Present × Unfavourable	0.001	-0.113	0.115	0.983
Present × Moderate	0.031	-0.025	0.087	0.277
**gMD**				
Present × Unfavourable	0.036	-0.072	0.144	0.509
Present × Moderate	-0.006	-0.058	0.047	0.836

Note: Standardised betas (β) and 95% confidence intervals are reported in fully adjusted models. Bold type indicates P<0.05; * indicates FDR significant ($p_{(FDR)}$ <0.05). gFrontal, general factors of frontal lobe volumes; gFA, general factors of fractional anisotropy; gMD, general factors of mean diffusivity; gICVF, general factors of intracellular volume fraction; gISOVF, general factors of isotropic volume fraction; gOD, general factors of orientation dispersion.

Table S8. Individual two-way interactions between *APOE* e4 genotype and each lifestyle factor, on brain structural phenotypes

Brain structural phenotypes		Standardised β	95% CI Lower	95% CI Upper	P values
**Smoke × *APOE* e4**					
Grey matter volume	Present × Current smoker	0.015	-0.038	0.067	0.579
	Present × Former smoker	-0.014	-0.034	0.005	0.146
White matter volume	Present × Current smoker	-0.016	-0.063	0.031	0.503
	Present × Former smoker	0.000	-0.018	0.017	0.982
Total brain volume	Present × Current smoker	-0.002	-0.022	0.019	0.882
	Present × Former smoker	-0.007	-0.015	0.000	0.058
Left hippocampus volume	Present × Current smoker	-0.136	-0.279	0.007	0.063
	Present × Former smoker	-0.035	-0.088	0.019	0.201
Right hippocampus volume	Present × Current smoker	-0.114	-0.256	0.028	0.116
	Present × Former smoker	-0.034	-0.087	0.019	0.206
White matter hyperintensity volume	Present × Current smoker	0.107	-0.026	0.240	0.116
	Present × Former smoker	0.037	-0.012	0.087	0.140
Total hippocampus volume	Present × Current smoker	-0.138	-0.276	0.001	0.051
	Present × Former smoker	-0.038	-0.089	0.013	0.148
Hippocampus asymmetry	Present × Current smoker	-0.021	-0.182	0.139	0.794
	Present × Former smoker	0.001	-0.059	0.060	0.986
gFrontal	Present × Current smoker	-0.001	-0.094	0.092	0.983
	Present × Former smoker	-0.004	-0.039	0.030	0.816
gICVF	Present × Current smoker	0.098	-0.057	0.252	0.216
	Present × Former smoker	-0.025	-0.083	0.032	0.391
gISOVF	Present × Current smoker	-0.018	-0.168	0.131	0.812
	Present × Former smoker	0.006	-0.049	0.062	0.820
gOD	Present × Current smoker	0.006	-0.145	0.157	0.941
	Present × Former smoker	0.003	-0.054	0.059	0.926
gFA	Present × Current smoker	0.098	-0.055	0.251	0.209
	Present × Former smoker	-0.026	-0.083	0.031	0.371
gMD	Present × Current smoker	-0.067	-0.211	0.078	0.367
	Present × Former smoker	0.052	-0.002	0.106	0.058
**Alcohol drinking × *APOE* e4**					
Grey matter volume	Present × High risk	0.008	-0.034	0.051	0.699
	Present × Moderate risk	-0.004	-0.023	0.016	0.716
White matter volume	Present × High risk	0.006	-0.032	0.045	0.752
	Present × Moderate risk	0.010	-0.008	0.027	0.275
Total brain volume	Present × High risk	0.008	-0.009	0.024	0.367
	Present × Moderate risk	0.004	-0.004	0.011	0.349
Left hippocampus volume	Present × High risk	**0.151**	**0.033**	**0.268**	**0.012**
	Present × Moderate risk	0.036	-0.017	0.090	0.181
Right hippocampus volume	Present × High risk	0.119	0.003	0.235	0.045
	Present × Moderate risk	0.028	-0.025	0.081	0.305
White matter hyperintensity volume	Present × High risk	-0.008	-0.117	0.102	0.889
	Present × Moderate risk	0.037	-0.013	0.087	0.144
Total hippocampus volume	Present × High risk	**0.148**	**0.035**	**0.262**	**0.010**
	Present × Moderate risk	0.035	-0.016	0.087	0.180
Hippocampus asymmetry	Present × High risk	0.032	-0.100	0.164	0.631
	Present × Moderate risk	0.009	-0.051	0.069	0.766
gFrontal	Present × High risk	0.073	-0.003	0.149	0.061
	Present × Moderate risk	-0.003	-0.038	0.032	0.861
gICVF	Present × High risk	-0.016	-0.143	0.111	0.802
	Present × Moderate risk	0.012	-0.046	0.070	0.687
gISOVF	Present × High risk	0.026	-0.096	0.148	0.677
	Present × Moderate risk	-0.009	-0.065	0.047	0.752
gOD	Present × High risk	0.081	-0.043	0.204	0.201
	Present × Moderate risk	**-0.056**	**-0.113**	**0.000**	**0.050**
gFA	Present × High risk	-0.039	-0.164	0.087	0.546
	Present × Moderate risk	0.042	-0.016	0.099	0.155
gMD	Present × High risk	0.029	-0.090	0.147	0.636
	Present × Moderate risk	-0.004	-0.058	0.050	0.886
**Diet × *APOE* e4**					
Grey matter volume	Present × Unhealthy diet	0.027	-0.007	0.061	0.119
	Present × Moderate diet	0.023	-0.006	0.051	0.120
White matter volume	Present × Unhealthy diet	-0.020	-0.051	0.010	0.194
	Present × Moderate diet	-0.023	-0.049	0.003	0.079
Total brain volume	Present × Unhealthy diet	0.002	-0.011	0.015	0.740
	Present × Moderate diet	-0.001	-0.013	0.010	0.795
Left hippocampus volume	Present × Unhealthy diet	-0.011	-0.104	0.081	0.811
	Present × Moderate diet	-0.034	-0.112	0.045	0.398
Right hippocampus volume	Present × Unhealthy diet	0.013	-0.078	0.105	0.774
	Present × Moderate diet	-0.015	-0.092	0.063	0.706

White matter hyperintensity volume	Present × Unhealthy diet	-0.045	-0.131	0.041	0.307
	Present × Moderate diet	-0.025	-0.098	0.048	0.496
Total hippocampus volume	Present × Unhealthy diet	0.001	-0.088	0.091	0.975
	Present × Moderate diet	-0.027	-0.102	0.049	0.489
Hippocampus asymmetry	Present × Unhealthy diet	-0.029	-0.133	0.074	0.580
	Present × Moderate diet	-0.021	-0.109	0.067	0.635
gFrontal	Present × Unhealthy diet	0.031	-0.029	0.091	0.316
	Present × Moderate diet	0.036	-0.014	0.087	0.161
gICVF	Present × Unhealthy diet	-0.026	-0.126	0.074	0.611
	Present × Moderate diet	-0.001	-0.086	0.083	0.973
gISOVF	Present × Unhealthy diet	0.018	-0.079	0.114	0.719
	Present × Moderate diet	0.002	-0.080	0.083	0.968
gOD	Present × Unhealthy diet	0.014	-0.083	0.111	0.777
	Present × Moderate diet	0.040	-0.043	0.122	0.347
gFA	Present × Unhealthy diet	-0.010	-0.109	0.089	0.844
	Present × Moderate diet	-0.017	-0.101	0.067	0.688
gMD	Present × Unhealthy diet	0.021	-0.073	0.114	0.665
	Present × Moderate diet	0.027	-0.052	0.106	0.498
**Sedentary behaviour × *APOE* e4**					
Grey matter volume	Present × High duration	-0.004	-0.028	0.019	0.718
	Present × Medium duration	-0.010	-0.031	0.012	0.382
White matter volume	Present × High duration	0.005	-0.017	0.026	0.673
	Present × Medium duration	0.007	-0.012	0.026	0.456
Total brain volume	Present × High duration	0.000	-0.009	0.010	0.937
	Present × Medium duration	-0.001	-0.009	0.008	0.869
Left hippocampus volume	Present × High duration	0.045	-0.020	0.110	0.175
	Present × Medium duration	-0.009	-0.067	0.050	0.775
Right hippocampus volume	Present × High duration	0.051	-0.014	0.115	0.123
	Present × Medium duration	-0.033	-0.091	0.025	0.264
White matter hyperintensity volume	Present × High duration	0.017	-0.044	0.077	0.592
	Present × Medium duration	**0.061**	**0.007**	**0.116**	**0.027**
Total hippocampus volume	Present × High duration	0.053	-0.010	0.116	0.099
	Present × Medium duration	-0.023	-0.079	0.033	0.422
Hippocampus asymmetry	Present × High duration	-0.009	-0.082	0.064	0.816
	Present × Medium duration	0.030	-0.036	0.095	0.374
gFrontal	Present × High duration	-0.022	-0.065	0.020	0.299
	Present × Medium duration	-0.012	-0.049	0.026	0.544
gICVF	Present × High duration	0.019	-0.051	0.090	0.594
	Present × Medium duration	-0.033	-0.096	0.030	0.303
gISOVF	Present × High duration	0.046	-0.022	0.114	0.185
	Present × Medium duration	-0.011	-0.071	0.050	0.729
gOD	Present × High duration	0.011	-0.058	0.079	0.764
	Present × Medium duration	0.022	-0.039	0.084	0.473
gFA	Present × High duration	-0.002	-0.072	0.068	0.950
	Present × Medium duration	-0.039	-0.101	0.024	0.226
gMD	Present × High duration	-0.003	-0.069	0.063	0.922
	Present × Medium duration	-0.001	-0.060	0.058	0.971
**Physical activity × *APOE* e4**					
Grey matter volume	Present × Low level	-0.008	-0.031	0.015	0.481
	Present × Medium level	-0.005	-0.027	0.017	0.666
White matter volume	Present × Low level	0.007	-0.013	0.028	0.490
	Present × Medium level	-0.006	-0.026	0.014	0.537
Total brain volume	Present × Low level	0.000	-0.009	0.009	0.986
	Present × Medium level	-0.006	-0.015	0.003	0.178
Left hippocampus volume	Present × Low level	-0.013	-0.075	0.049	0.680
	Present × Medium level	-0.005	-0.066	0.057	0.879
Right hippocampus volume	Present × Low level	0.004	-0.057	0.065	0.896
	Present × Medium level	-0.042	-0.103	0.018	0.172
White matter hyperintensity volume	Present × Low level	-0.016	-0.074	0.041	0.582
	Present × Medium level	0.011	-0.046	0.068	0.697
Total hippocampus volume	Present × Low level	-0.005	-0.065	0.055	0.876
	Present × Medium level	-0.026	-0.085	0.033	0.384
Hippocampus asymmetry	Present × Low level	-0.020	-0.089	0.049	0.571
	Present × Medium level	0.045	-0.023	0.114	0.196
gFrontal	Present × Low level	-0.002	-0.042	0.038	0.913
	Present × Medium level	-0.018	-0.058	0.022	0.370
gICVF	Present × Low level	0.031	-0.036	0.097	0.370
	Present × Medium level	0.004	-0.062	0.070	0.909
gISOVF	Present × Low level	0.060	-0.005	0.124	0.070
	Present × Medium level	**0.068**	**0.004**	**0.132**	**0.036**
gOD	Present × Low level	-0.006	-0.071	0.059	0.859
	Present × Medium level	0.006	-0.058	0.071	0.850

gFA	Present × Low level	0.032	-0.034	0.099	0.337
	Present × Medium level	0.004	-0.062	0.070	0.905
gMD	Present × Low level	0.015	-0.047	0.078	0.628
	Present × Medium level	0.041	-0.021	0.103	0.198

Note: Standardised betas (β) and 95% confidence intervals are reported in fully adjusted models. Bold type indicates P<0.05; * indicates FDR significant ($p_{(FDR)}$ <0.05). gFrontal, general factors of frontal lobe volumes; gICVF, general factors of intracellular volume fraction; gISOVF, general factors of isotropic volume fraction; gOD, general factors of orientation dispersion; gFA, general factors of fractional anisotropy; gMD, general factors of mean diffusivity.

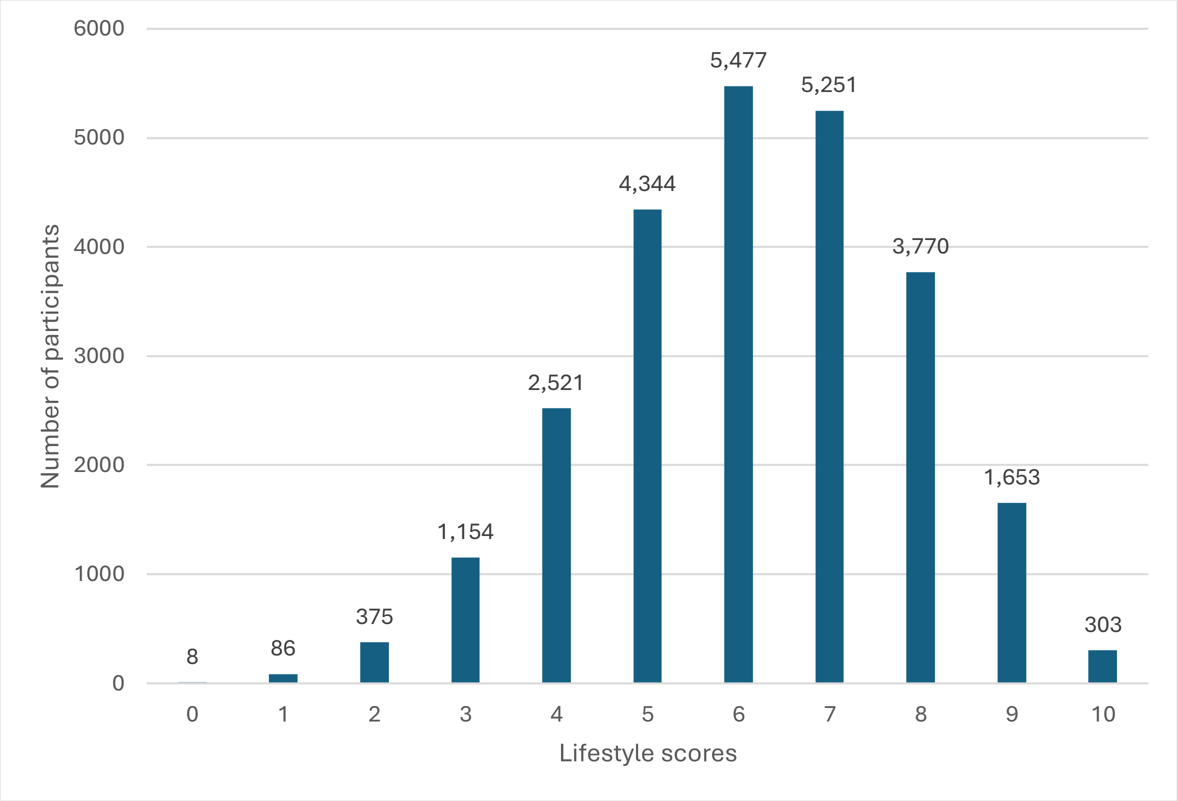

Figure S1. Histogram showing the distribution of lifestyle scores.

Note: Participants who scored 0, 1, 2 or 3 were classed as unfavourable lifestyle; 4, 5, or 6 as moderate lifestyle; 7, 8, 9, or 10 as favourable lifestyle (N=24912).

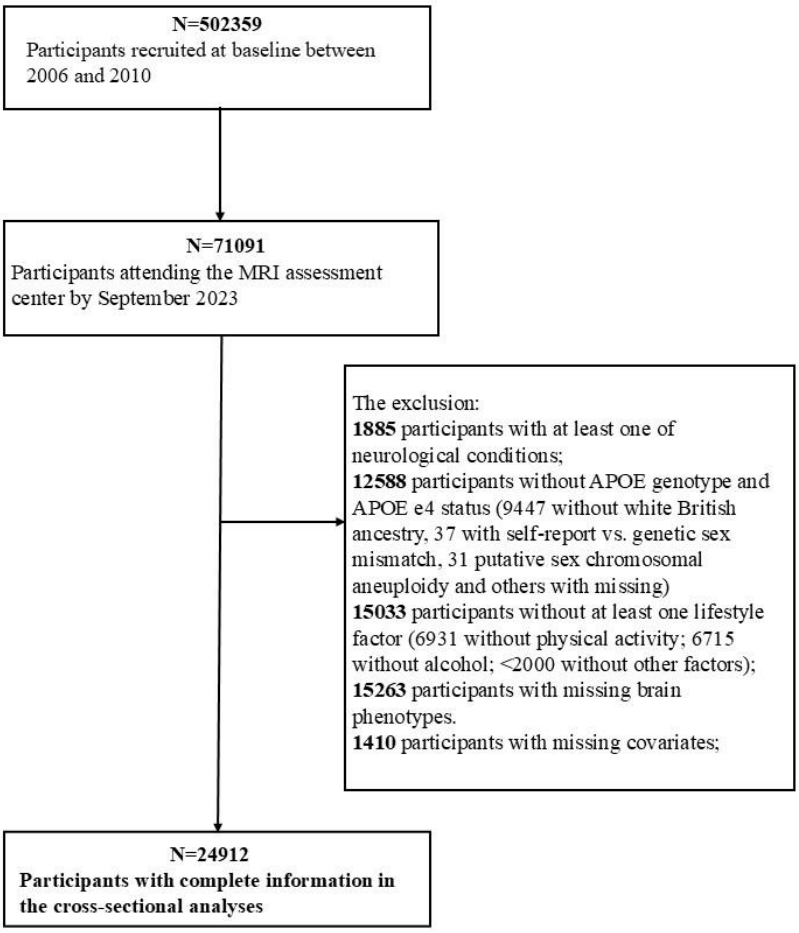

Figure S2. Flowchart summarising participants selection and inclusion processes, including the reasons for exclusion of participants.
